# Neutrophil cell death in response to infection and its relation to coagulation

**DOI:** 10.1186/2052-0492-1-13

**Published:** 2013-12-04

**Authors:** Toshiaki Iba, Naoyuki Hashiguchi, Isao Nagaoka, Yoko Tabe, Miwa Murai

**Affiliations:** Department of Emergency and Disaster Medicine, Juntendo University, 2-1-1 Hongo, Bunkyo-ku, Tokyo, 113-8421 Japan; Department of Defense and Biochemical Research, Juntendo University, 2-1-1 Hongo, Bunkyo-ku, Tokyo, 113-8421 Japan; Department of Clinical Laboratory Medicine, Juntendo University, 2-1-1 Hongo, Bunkyo-ku, Tokyo, 113-8421 Japan

**Keywords:** Neutrophil extracellular traps, NETosis, Apoptosis, Necrosis

## Abstract

Neutrophil is a major player in the pathophysiology of severe sepsis. Recent studies have revealed that the cell death mechanism of neutrophils directly relates to the development of organ dysfunction during sepsis. Here we discuss about the different types of neutrophil cell death such as necrosis, apoptosis, autophagy, and the unique cell death style dubbed NETosis. NETosis cells release neutrophil extracellular traps (NETs), which are composed of chromatin bound to granular and nucleic proteins. The primary purpose of NET release is thought to be the control of microbial infections; however, it acts as a danger signal for the host as well. The harmful substances such as DNA, histones, and high-mobility group box 1 (HMGB1) and many other danger-associated molecular patterns (DAMPs) released along with NETosis or from necrotic neutrophils also contribute to the pathogenesis of sepsis. At the same time, the coagulation system, which is closely tied to these neutrophil cell death mechanisms, is often over-activated. It is well known that individual bacterial pathogens express virulence factors that modulate cell death pathways and influence the coagulation disorder during sepsis. Moreover, extensive cross talk exists between these two phenomena, whereby inflammation leads to activation of coagulation and coagulation considerably affects inflammatory activity. A greater knowledge of cell death pathways in sepsis informs the potential for future therapies designed to ameliorate excessive immune responses during sepsis.

## Introduction

Neutrophils play a central role in the innate immune system [1]. The well-known functions of neutrophils are the migration to the infection site where they engulf and inactivate microorganisms through the fusion of phagosomes with granules and the formation of phagolysosomes, in which antimicrobial enzymes and reactive oxygen species (ROS) act synergistically for the clearance of pathogens [2]. Excessive neutrophil activation results in degranulation and release of ROS into the extracellular medium, which leads to host tissue injury [3], while neutrophil apoptosis contributes to the resolution of inflammation [4].

Neutrophil cell death is divided into necrosis, apoptosis (type 1 programmed cell death), autophagy (type 2 programmed cell death), the newly recognized NETosis, and some other types. Necrosis is characterized as cell death exhibiting cytoplasmic swelling, disorganized organelles, ruptured plasma membrane, and lytic nucleus [5]. In contrast, chromatin condensation is the notable feature of apoptosis, which later proceeds to the fragmentation of the nucleus and formation of apoptotic bodies consisting of intact plasma membrane and various organelles. Autophagy is known as a process of degradation of the self-components with a purpose of recycling cytoplasmic components. It is characterized by the formation of autophagosomes, which are large vesicles containing cytosol and organelle, which then fuse with lysosomes and are degraded without cell damage [6]. As a matter of fact, these forms of cell death appear to be transformable. For example, autophagy is thought to be a reversible cell death; however, once autophagic capacity is overwhelmed by certain stimulations, apoptosis is triggered. Apoptotic cells often turn to necrosis if the stimulus is too strong, and this type of cell death is known as aponecrosis.

The fourth style of neutrophil cell death NETosis is quite unique. NETosis is a type of programmed cell death. Neutrophils can kill pathogens extracellularly by releasing neutrophil extracellular traps (NETs) [7]. The impact of NETs derives from the combined antimicrobial activities of chromatin, histone, elastase, and other cytoplasmic proteins. In this review, we introduce the mechanism and the morphological findings of major types of neutrophil deaths in the following part.

## Review

### Neutrophil function against microbial infection

**Figure 1:**
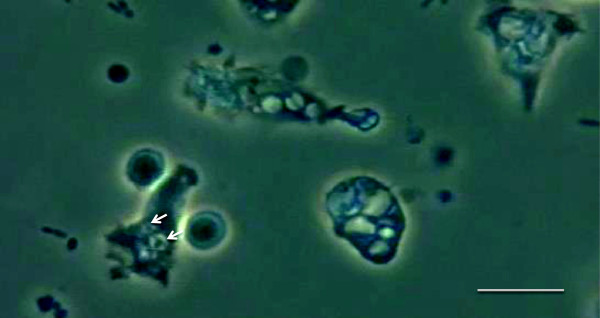
**Phase-contrast microscopic view of phagocytosis (objective × 40).** Phagocytosed *Escherichia coli* are visualized inside vacuoles known as phagolysosomes (*arrows*). *Scale bar* represents 10-μm length.

Neutrophils protect the body at the front line against microbial infection. Once the bacterial or fungal infection is established, neutrophils are discharged from the bone marrow into the bloodstream within hours and then migrate into the extravascular infectious site following the chemoattractants. Tissue neutrophils are activated by the inflammatory cytokines, complements, and other pro-inflammatory mediators. At the infectious site, neutrophils ingest and digest microorganisms, which is known as phagocytosis, and this process has been thought to be the most important role of neutrophils in the host defense. Neutrophils phagocytose and engulf microbes into phagosomes that rapidly fuse with the granules which contain the toxic molecules, phospholipases, ROS, and proteases including lysozyme, bactericidal permeability-increasing protein (BPI), defensins, and cathelicidins [8] (Figure 1).

Neutrophils are inherently short-lived, approximately 5–6 days, and undergo spontaneous apoptosis [9]. In infected tissues, their apoptosis can be delayed both by microbial constituents and by pro-inflammatory stimuli [10, 11]. Generally, the tissue neutrophils die in apoptosis; however, if the infection is serious enough, some undergo necrosis or other styles of cell death. Except for apoptotic and autophagic cell deaths, uncontrolled release of toxic substances from the dead neutrophils can propagate the inflammatory response leading to tissue damage. Therefore, avoidance of unprogrammed death and scavenging of the dying neutrophils is crucial for the maintenance of homeostasis [12, 13]. To terminate the inflammation, it is necessary not only to attenuate the generation of anti-inflammatory mediators but also to remove the inflammatory cells along with the microbes they have ingested [14].

### Necrosis

#### Induction of necrosis

Most neutrophils undergo apoptosis after they leave the peripheral circulation without infection [15]. When apoptosis proceeds in an orderly fashion, tissue macrophages and other phagocytes ingest the apoptotic bodies which include potentially injurious granular enzymes. In contrast, necrosis is a turbulent cell death. If this accidental cell death is triggered by unexpected events, toxic constituents including proteolytic enzymes and oxidant-generating enzymes are released from the necrotic cells in an unregulated manner. Neutrophil necrosis is probably one of the major causes of tissue damage during infection [16], but little is known as to how they undergo necrosis, and there is no simple method that can detect the neutrophils undergoing necrosis.

#### Response to necrosis

Necrotic cells release a variety of danger signals known as damage-associated molecular patterns (DAMPs) such as high-mobility group box 1 (HMGB1), uric acid, heat shock proteins, DNA-chromatin complexes, and antimicrobial peptides. Many of these substances are recognized by specific receptors named pattern-recognizing receptors (PRRs) and stimulate the synthesis of pro-inflammatory mediators. For example, HMGB1, a nuclear protein binding to DNA and regulating gene transcription, is released from the necrotic cells and has been shown to stimulate inflammatory cytokine secretion by monocytes [17]. Uric acid and its active form, monosodium urate (MSU), are released to the cytosolic compartment under inflammatory stimulation. MSU has recently attracted attention as a strong inducer of inflammatory reaction [18]. DNA-chromatin complexes [19] and heat shock proteins [20, 21] have also been shown to stimulate pro-inflammatory cytokine production [22]. Since PRRs have been known to recognize the molecular patterns of microorganisms and their related products, the intra- and extrainflammatory stimulus known as pathogen-associated molecular patterns (PAMPs) in sepsis is mediated through similar receptors. PAMPs are common components to many microbes, for example, lipopolysaccharide, peptidoglycan, and flagellin, which are of bacterial origin, as well as RNA and DNA, which can be of viral or bacterial origin. As for PRRs, Toll-like receptor (TLR) is the best known, and more than ten subtypes have been identified in humans. Among them, TLR-3 is a receptor for viral double-stranded RNA, which allows macrophages to recognize by-products of necrotic neutrophils, thereby stimulating the generation of pro-inflammatory cytokines [23].

#### The appearance of necrotic neutrophils

**Figure 2:**
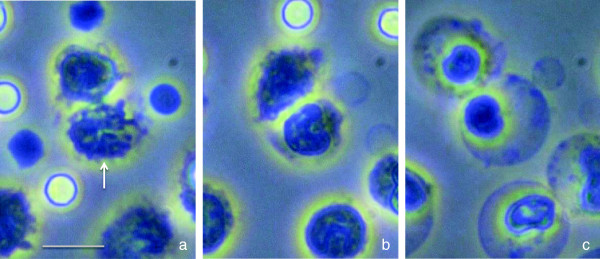
**Morphological changes in necrotic neutrophils.** Time-lapse images of the neutrophils. Necrosis was induced by lipopolysaccharide *in vitro*. The lobulated nucleus (*arrow*) **(a)** fused and became a round-like mononuclear cell **(b)**. The necrotic cells terminally compromise the cellular membrane that allows cells to swell **(c)**. *Scale bar* represents 10-μm length.

**Figure 3:**
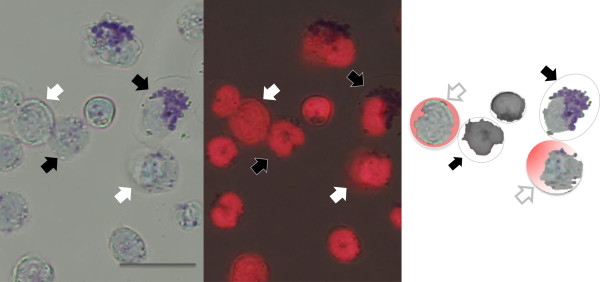
**Immunofluorescent staining of necrotic neutrophils.** Bright-field (*left panel*) and overlay (*middle panel*) images showing DNA distribution in mouse neutrophils stimulated by lipopolysaccharide. In the necrotic neutrophils (*white arrows*), DNA spreads in the cytoplasm and are stained by ID-red (*red*), while other cells keep the granules and DNA resides in nuclei (*black arrows*). The *right panel* shows the combined image of the selected cells. Microscopic pictures were taken 3 h after stimulation. All *scale bars* represent 20-μm length.

Necrotic neutrophils are specified to have a swollen cytoplasm with an undisposed nucleus. Under serial observation, the initial step of the morphological change was observed in the nucleus, i.e., lobulated nuclei (Figure 2a) fused and turned to a large round structure (Figure 2b). In the second step, ballooning of the cell and membrane disintegration were recognized (Figure 2c). However, these were not the primary morphological changes since many of the neutrophils undergo apoptosis initially. But, if the insult is severe enough, some neutrophils under the pathway of apoptosis turns to necrosis, and this style is known as ‘secondary necrosis’ [24]. At the late stage of necrosis, although the nuclear membrane appeared intact, the chromatin had decondensed and the nuclear contents have spilled out into the cytoplasm, and this event was recognized as the staining of the cytoplasm under the immunofluorescence microscopic observation (Figure 3, middle). Cell necrosis finally leads to the permeabilization of the cytoplasmic membrane and cell disintegration (open autolysis) with leakage of cell contents [25].

### Apoptosis

#### Induction of apoptosis

In contrast to necrosis, which is a passive sequence of events leading to disintegration of the nuclear envelope and cytoplasmic membranes, apoptosis represents a highly organized programmed cell death [26]. The ability to eliminate cells by apoptosis rather than necrosis is favorable to the host because the cell elimination in an orderly manner can limit the extent of cell death and inflammation caused by the uncontrolled release of toxic neutrophil products during necrosis. Apoptosis is initiated by either an intrinsic or an extrinsic pathway. The intrinsic pathway is activated by different noxious stimuli and involves the release of cytochrome C from mitochondria into the cytosol [27]. This release triggers the activation of intracellular caspases responsible for the cleavage of DNA and structural cytoplasmic proteins [28]. The second, extrinsic pathway of apoptosis is triggered by the binding of extracellular ligands such as tumor necrosis factor (TNF)-α or Fas ligand (FasL) to specific TNF receptors on the cell surface [29]. The binding of these ligands then generates a transmembrane signal to activate the caspase sequence. In the case of neutrophil, the timing of apoptosis is strictly regulated. Neutrophil granulocytes in particular are primed to undergo apoptosis within 24–48 h after having left the systemic circulation, but the exact time at which this occurs is influenced by several factors [30, 31].

#### The appearance of apoptotic neutrophils

**Figure 4:**
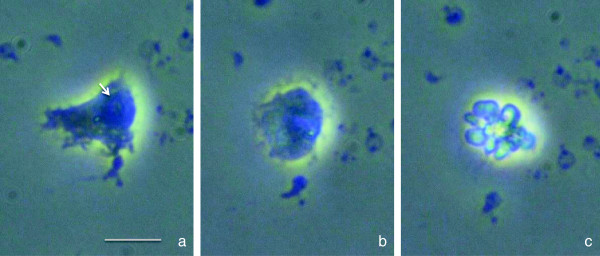
**Morphological changes in apoptotic neutrophils.** Time-lapse images of the neutrophils. Early apoptotic morphology with a condensed nucleus induced by lipopolysaccharide (*arrow*) **(a)**. Roundup cell with perinuclear chromatin aggregation but cytoplasm integrity **(b)**. At the late stage of apoptosis, the nucleus collapses and cytoplasmic granules are tightly packed. Finally, blebs are formed by the plasma membrane **(c)**. *Scale bar* represents 10-μm length.

Having fulfilled their purpose at a site of infection, neutrophils undergo apoptosis and efficiently dispose cells through ingestion by macrophages [32, 33]. Morphological features of apoptosis include the condensation of chromatin (Figure 4a) and its migration to the nuclear periphery (Figure 4b), fragmentation of nuclear DNA, and the blebbing of cell membranes, forming apoptotic bodies (Figure 4c) ready for ingestion by the neighboring phagocytes [32, 34].

### Autophagy

Autophagy is a homeostatic mechanism involved in the clearance of damaged organelles and in cellular survival under certain stresses or nutrient depletion to provide essential nutrients and proteins through recycling of the cytosolic organelles [35]. The implication of autophagy in innate immunity is yet to be clarified, but one possible explanation for activated autophagy during sepsis is that the autophagic process results in the removal of intracellular pathogens (xenophagy) during sepsis [36, 37]. The regulation of autophagy by activation of ROS, TLR, and inflammatory cytokines such as TNF-α and interferons has been reported [38–40]. TLR activation does not result in excess oxidative burst, but the inhibition of apoptosis leads to the induction of autophagy [41, 42]. Considering the regulatory role of apoptosis in the inflammatory process, the pro-survival induction of autophagy in neutrophils enhances the inflammatory responses by delaying cell death and could be involved in the pathogenesis of sepsis related to suppression of apoptosis, leading to tissue injury. As mentioned previously, autophagy is basically the survival mechanism of cells; however, when the insult is excessive, autophagy results in autophagic cell death. Morphological features of this cell death include vacuolization, degradation of cytoplasmic content, and lack of chromatin condensation. Cells undergoing autophagic cell death may be internalized by neighboring cells. Therefore, this type of cell death is considered to be a noninflammatory form [43].

### NETosis

#### Induction of NETosis

The term ‘NETosis’ for neutrophil cell death leads to the formation of NETs. NETosis is the third programmed neutrophil cell death, which is quite different from other types of cell death. Under the circumstances of bacterial, fungal, or parasitic infection, microbial components such as lipopolysaccharide and lipoteichoic acid, and ROS including hydrogen peroxide, can induce peculiar morphological changes in neutrophils [44]. Rapid NET formation is also induced by platelets activated via TLR-4 [45]. Similarly, alarmines such as heat shock proteins, HMGB1, as well as RNA and DNA of host origin are detected as the initiators of NETosis. With regard to PRRs, RNA and DNA are reported to be sensed by TLR-9 [46], while histones are reported to be sensed through TLR-2 and TLR-4 [47].

#### Mechanisms of NETosis

Under a certain stimulation, ROS is activated as the first step, then neutrophil elastase (NE) and myeloperoxidase (MPO) migrate from granules to the nucleus, and finally, the processing of histones leads to rupture of the cell. One of the most common inducers of NETosis is phorbol myristate acetate (PMA), which directly stimulates protein kinase C (PKC) and subsequently leads to the production of ROS. One of the distinctive appearances of NETosis is the homogenous nucleoplasm, and this change depends on the activity of NE and MPO. NE is initially stored in azurophilic granules in the cytosol. After the stimulation, NE is released from the granules and enters the nucleus, where it degrades the linker histone H1 and processes core histones [48]. MPO also migrates to the nucleus and it enhances chromatin decondensation. Thus, NE and MPO cooperate to undergo further histone modifications to decondense the chromatin structure. Eventually, NETs are quickly removed once the infection is resolved. NETs are susceptible to DNase1 [49], and the debris left by DNase1 will be cleared by macrophages and neutrophils recruited to the inflammatory site [50].

#### The role of NETs

One of the major roles of neutrophils is the elimination of microorganisms. For that purpose, NETs are expected to trap microbes and prevent their dissemination to the circulating blood. Inactivation of the virulence factors and the extermination of pathogens are also requested. Trapping microbes, most likely through charge interaction [51], prevents their dissemination and encloses them at the initial site of infection. Interestingly, Group A *Streptococcus pyogenes*[52], pneumococcus, and *Staphylococcus aureus*[53] are capable of liberating themselves from NETs since they encode endonucleases. As a matter of fact, the expression of DNase is essential for these bacteria to be pathogenic [52]. Other than DNA, NETs contain several proteins toxic to microbes. These include lysozyme, antimicrobial peptides, ion chelators (calgranulin), and histones. The antimicrobial activity of NETs is likely the result of the combination of these components. Their effects are enhanced by the combination work and the high local concentrations achieved on NETs. MPO on NETs is also essential to kill microbes. The antifungal activity of NETs has been assigned to calgranulin [51]. Histones are the main toxic components of NETs; however, the mechanism of histone toxicity is poorly understood. In severe sepsis, extranuclear histones can be detected in circulating blood, which are released abundantly during NETosis [51]. Since circulating histones are also harmful for the host cells [54], histones are the target of the new therapeutic strategy.

#### The appearance of NETs

**Figure 5:**
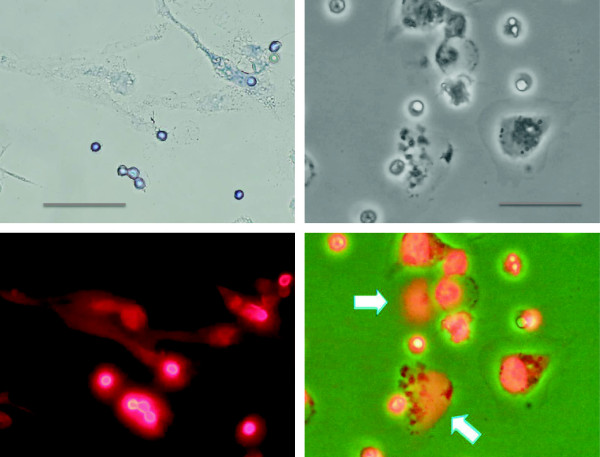
**Immunofluorescent staining of NETs.** The bright-field (*left upper panel*) view of NETosis induced by 10 nM of PMA. Immunofluorescent staining by propidium iodine visualizes DNA ejected from the neutrophils (*left bottom panel*, same field as above). Phase-contrast view of the neutrophils stimulated by PMA (*right upper panel*) and the overlay image (*right bottom panel*) showing DNA distribution (*red*). *Arrows* indicate the expelled DNA. All *scale bars* represent 20-μm length.

**Figure 6:**
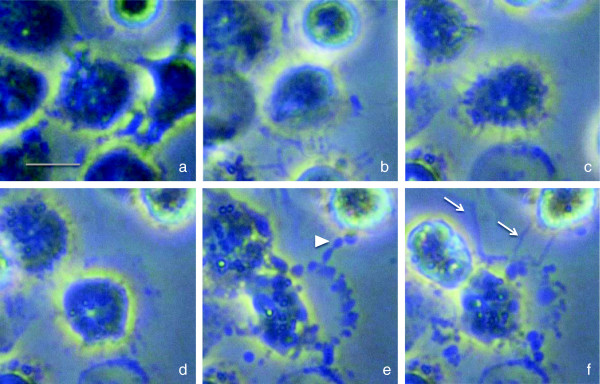
**Morphological changes in NETosis.** Time-lapse images of the neutrophils. The nucleus loses its lobules **(a)**, the chromatin decondenses **(b)**, the nuclear envelope disaggregates **(c)**, the nucleoplasm becomes homogenous **(d)**, the cell membrane ruptures and the interior of the cell is ejected (*arrowhead*) **(e)**, and chromatins (*arrows*) are expelled **(f)**. NETosis was induced by 10 nM of PMA. *Scale bar* represents 10-μm length.

NETs are hardly seen on light microscopy. They just look like debris of the dead cells (Figure 5, left upper panel). In general, NETosis is morphologically characterized by the loss of intracellular membranes before the integrity of the plasma membrane is compromised [55]. The structure of NETs observed by electron microscopy is quite unique; NETs consist of net-like filaments of nucleosomes with a diameter of approximately 17 nm and stud-like components of granular proteins with a diameter of approximately 50 nm [51]. This morphology in scanning electron microscopy easily differentiates NETs from other fibrous structures such as fibrin. Under immunofluorescent microscopy, NETs are visualized as cloud-like structures surrounding the dead neutrophils (Figure 5, lower panels). The time-course of NETosis is as follows: minutes after activation, neutrophils flattened and firmly attached to the substratum; during the next hour, the nucleus loses its lobules (Figure 6a) and the chromatin decondenses (Figure 6b); after several hours, the nuclear envelope disaggregates into vesicles (Figure 6c) and the nucleoplasm become homogenous (Figure 6d); and finally, the cell membrane ruptures and the interior of the cell is ejected into the extracellular space (Figure 6e), forming NETs (Figure 6f).

### Neutrophil cell death and coagulation

There is universal agreement that dysfunction in coagulation develops during sepsis and leads to inappropriate intravascular thrombus formation. In contrast, discussions are still going on whether coagulopathy has pathogenic roles in the progress of sepsis or is a mere response to the insult. This debate is still inconclusive since the results of the clinical trials using anticoagulants are inconsistent [56–59].

**Figure 7:**
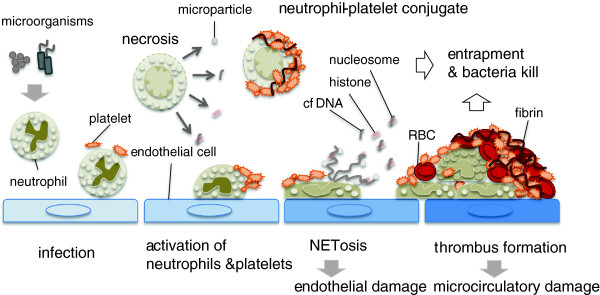
**NETosis and microthrombus formation in sepsis.** Microbes activate the neutrophils. Activated neutrophils activate platelets and the coagulation system by expelling microparticles, nuclear components, and granular proteins. At the same time, neutrophils accumulate and adhere to the endothelium in collaboration with platelets during sepsis. There, neutrophils expel NETs that kill bacteria and activate the coagulation system. NETs serve as a scaffold for thrombus formation. Thrombi substantially lead to microcirculatory damage and then organ dysfunction in sepsis.

One of the purposes of this review is to elucidate that neutrophil death relates to the activation of coagulation. Indeed, the fact that activation of the coagulation system represents an essential innate immune response that limits microbial spreading is a global consensus [60]. Monocytes/macrophages are widely accepted as the main players in the pro-coagulant process; however, recent evidences have suggested that neutrophils also play important roles. One of the mechanisms is explained by the tissue factor exposed on the surface of the dying neutrophils [61], as well as on microparticles derived from neutrophils [62]. Neutrophil-derived proteinases such as elastase and cathepsin G from the dead neutrophils are another contributor. These proteases cleave tissue factor pathway inhibitor (TFPI) [63] and anticoagulants such as antithrombin and activated protein C [64]. Other than these sources, NETs also provide pro-coagulant activities. During sepsis, neutrophils accumulate and adhere tightly to the endothelium. There, neutrophils expel NETs that serve as a scaffold for thrombus formation [65] (Figure 7). Serine proteases in neutrophil, such as NE and cathepsin G, degrade physiological coagulation inhibitors such as antithrombin and accelerate coagulation. The major components of NETs, chromatin and histones, are the strong initiators of coagulation [54, 66]. These phenomena indicate that clot formation is enhanced by NETs. Other than those, NETs express high amounts of tissue factor. The release of tissue factor-bearing NETs at the sites of inflammation may result in the localized activation of the coagulation cascade [67]. We now know that numerous neutrophils are recruited and adhere to the vascular endothelium and play major roles in thrombus formation. There, activated platelets cooperate with neutrophils to form NETs as well [68, 69].

Thrombus formation causes obstruction in the microvasculature and induces tissue ischemia and injury, thereby contributing to multiple organ failure and death. Therefore, if microthrombus formation is prevented or shut off, the organ function should be preserved. But, in practice, there are multilayered confounders in such settings, including population heterogeneity, comorbidities, and contraindications to standard care therapeutics. Moreover, as stated in this review, since thrombus formation is an essential part of the host defense mechanism, neither unconditional application of anticoagulant therapy nor modulation of neutrophil death will be beneficial to the host.

## Conclusions

Neutrophil cell death plays a pivotal role in the pathophysiology of sepsis. Although apoptosis has been the major focus of investigation, necrosis, autophagic cell death, and the new comer NETosis also play significant roles in this critical situation. Actually, a variety of styles of cell death coexist in sepsis, and the population of necrosis and NETosis are especially important to regulate the host condition. The specific virulence factors expressed by individual pathogens, the degree of insult, the duration of stimulation, and the response of the host immune system may determine the distribution of cell death phenotypes. It is apparent that cell disintegration and molecules such as histones, nucleosomes, proteases, and tissue factor released from the dead cells all play governing roles in the inflammatory and coagulatory responses, therefore host immune competence, during sepsis.

## Abbreviations

BPI: Bactericidal permeability-increasing protein
DAMPs: Damage-associated molecular patterns
FasL: Fas ligand
HMGB1: High-mobility group box 1
MPO: Myeloperoxidase
MSU: Monosodium urate
NE: Neutrophil elastase
NETs: Neutrophil extracellular traps
PAMPs: Pathogen-associated molecular patterns
PKC: Protein kinase C
PMA: Phorbol myristate acetate
PRRs: Pattern-recognizing receptors
ROS: Reactive oxygen species
TLR: Toll-like receptor.

## References

[1] Nathan C (2006). Neutrophils and immunity: challenges and opportunities. Nat Rev Immunol.

[2] Mayer-Scholl A, Averhoff P, Zychlinsky A (2004). How do neutrophils and pathogens interact?. Curr Opin Microbiol.

[3] Weiss SJ (1989). Tissue destruction by neutrophils. N Engl J Med.

[4] Serhan CN, Savill J (2005). Resolution of inflammation: the beginning programs the end. Nat Immunol.

[5] Yasuhara S, Asai A, Sahani ND, Martyn JA (2007). Mitochondria, endoplasmic reticulum, and alternative pathways of cell death in critical illness. Crit Care Med.

[6] Klionsky DJ (2004). Cell biology: regulated self-cannibalism. Nature.

[7] Brinkmann V, Reichard U, Goosmann C, Fauler B, Uhlemann Y, Weiss DS, Weinrauch Y, Zychlinsky A (2004). Neutrophil extracellular traps kill bacteria. Science.

[8] Ganz T, Weiss J (1997). Antimicrobial peptides of phagocytes and epithelia. Semin Hematol.

[9] Haslett C (1997). Granulocyte apoptosis and inflammatory disease. Br Med Bull.

[10] Colotta F, Re F, Polentarutti N, Sozzani S, Mantovani A (1992). Modulation of granulocyte survival and programmed cell death by cytokines and bacterial products. Blood.

[11] Lee A, Whyte MK, Haslett C (1993). Inhibition of apoptosis and prolongation of neutrophil functional longevity by inflammatory mediators. J Leukoc Biol.

[12] Savill JS, Wyllie AH, Henson JE, Walport MJ, Henson PM, Haslett C (1989). Macrophage phagocytosis of aging neutrophils in inflammation. Programmed cell death in the neutrophil leads to its recognition by macrophages. J Clin Invest.

[13] Haslett C, Savill JS, Whyte MK, Stern M, Dransfield I, Meagher LC (1994). Granulocyte apoptosis and the control of inflammation. Philos Trans R Soc Lond B Biol Sci.

[14] Erwig LP, Henson PM (2007). Immunological consequences of apoptotic cell phagocytosis. Am J Pathol.

[15] Haslett C (1999). Granulocyte apoptosis and its role in the resolution and control of lung inflammation. Am J Respir Crit Care Med.

[16] Rydell-Tormanen K, Uller L, Erjefalt JS (2006). Direct evidence of secondary necrosis of neutrophils during intense lung inflammation. Eur Respir J.

[17] Scaffidi P, Misteli T, Bianchi ME (2002). Release of chromatin protein HMGB1 by necrotic cells triggers inflammation. Nature.

[18] Martinon F, Petrilli V, Mayor A, Tardivel A, Tschopp J (2006). Gout-associated uric acid crystals activate the NALP3 inflammasome. Nature.

[19] Hefeneider SH, Cornell KA, Brown LE, Bakke AC, McCoy SL, Bennett RM (1992). Nucleosomes and DNA bind to specific cell-surface molecules on murine cells and induce cytokine production. Clin Immunol Immunopathol.

[20] Tsan MF, Gao B (2004). Cytokine function of heat shock proteins. Am J Physiol Cell Physiol.

[21] Wallin RP, Lundqvist A, More SH, von Bonin A, Kiessling R, Ljunggren HG (2002). Heat-shock proteins as activators of the innate immune system. Trends Immunol.

[22] Zanetti M (2004). Cathelicidins, multifunctional peptides of the innate immunity. J Leukoc Biol.

[23] Cavassani KA, Ishii M, Wen H, Schaller MA, Lincoln PM, Lukacs NW, Hogaboam CM, Kunkel SL (2008). TLR3 is an endogenous sensor of tissue necrosis during acute inflammatory events. J Exp Med.

[24] Silva MT (2010). Bacteria-induced phagocyte secondary necrosis as a pathogenicity mechanism. J Leukoc Biol.

[25] Perry FE, Elson CJ, Mitchell TJ, Andrew PW, Catterall JR (1994). Characterisation of an oxidative response inhibitor produced by Streptococcus pneumoniae. Thorax.

[26] Pinheiro Da Silva F, Nizet V (2009). Cell death during sepsis: integration of disintegration in the inflammatory response to overwhelming infection. Apoptosis.

[27] Maianski NA, Geissler J, Srinivasula SM, Alnemri ES, Roos D, Kuijpers TW (2004). Functional characterization of mitochondria in neutrophils: a role restricted to apoptosis. Cell Death Differ.

[28] Wang X (2001). The expanding role of mitochondria in apoptosis. Genes Dev.

[29] Schulze-Osthoff K, Ferrari KD, Los M, Wesselborg S, Peter ME (1998). Apoptosis signaling by death receptors. Eur J Biochem.

[30] Akgul C, Moulding DA, Edwards SW (2001). Molecular control of neutrophil apoptosis. FEBS Lett.

[31] Simon HU (2003). Neutrophil apoptosis pathways and their modifications in inflammation. Immunol Rev.

[32] Hengartner MO (2000). The biochemistry of apoptosis. Nature.

[33] Savill J, Fadok V (2000). Corpse clearance defines the meaning of cell death. Nature.

[34] Fadok VA, Voelker DR, Campbell PA, Cohen JJ, Bratton DL, Henson PM (1992). Exposure of phosphatidylserine on the surface of apoptotic lymphocytes triggers specific recognition and removal by macrophages. J Immunol.

[35] Klionsky DJ, Emr SD (2000). Autophagy as a regulated pathway of cellular degradation. Science.

[36] Levine B, Deretic V (2007). Unveiling the roles of autophagy in innate and adaptive immunity. Nat Rev Immunol.

[37] Schmid D, Dengjel J, Schoor O, Stevanovic S, Munz C (2006). Autophagy in innate and adaptive immunity against intracellular pathogens. J Mol Med.

[38] Djavaheri-Mergny M, Amelotti M, Mathieu J, Besançon F, Bauvy C, Souquère S, Pierron G, Codogno P (2006). NF-κB activation represses tumor necrosis factor-α-induced autophagy. J Biol Chem.

[39] Tallóczy Z, Jiang W, Virgin HW, Leib DA, Scheuner D, Kaufman RJ, Eskelinen EL, Levine B (2002). Regulation of starvation- and virus-induced autophagy by the eIF2α kinase signaling pathway. Proc Natl Acad Sci USA.

[40] Gutierrez MG, Master SS, Singh SB, Taylor GA, Colombo MI, Deretic V (2004). Autophagy is a defense mechanism inhibiting BCG and Mycobacterium tuberculosis survival in infected macrophages. Cell.

[41] Hayashi F, Means TK, Luster AD (2003). Toll-like receptors stimulate human neutrophil function. Blood.

[42] François S, El Benna J, Dang PM, Pedruzzi E, Gougerot-Pocidalo MA, Elbim C (2005). Inhibition of neutrophil apoptosis by TLR agonists in whole blood: involvement of the phosphoinositide 3-kinase/Akt and NF-kappaB signaling pathways, leading to increased levels of Mcl-1, A1, and phosphorylated Bad. J Immun.

[43] Labbé K, Saleh M (2008). Cell death in the host response to infection. Cell Death Differ.

[44] Fuchs TA, Abed U, Goosmann C, Hurwitz R, Schulze I, Wahn V, Weinrauch Y, Brinkmann V, Zychlinsky A (2007). Novel cell death program leads to neutrophil extracellular traps. J Cell Biol.

[45] Clark SR, Ma AC, Tavener SA, McDonald B, Goodarzi Z, Kelly MM, Patel KD, Chakrabarti S, McAvoy E, Sinclair GD, Keys EM, Allen-Vercoe E, Devinney R, Doig CJ, Green FH, Kubes P (2007). Platelet TLR4 activates neutrophil extracellular traps to ensnare bacteria in septic blood. Nat Med.

[46] Garcia-Romo GS, Caielli S, Vega B, Connolly J, Allantaz F, Xu Z, Punaro M, Baisch J, Guiducci C, Coffman RL, Barrat FJ, Banchereau J, Pascual V (2010). Netting neutrophils are major inducers of type I IFN production in pediatric systemic lupus erythematosus. Sci Transl Med.

[47] Semeraro F, Ammollo CT, Morrissey JH, Dale GL, Friese P, Esmon NL, Esmon CT (2011). Extracellular histones promote thrombin generation through platelet-dependent mechanisms: involvement of platelet TLR2 and TLR4. Blood.

[48] Papayannopoulos V, Metzler KD, Hakkim A, Zychlinsky A (2010). Neutrophil elastase and myeloperoxidase regulate the formation of neutrophil extracellular traps. J Cell Biol.

[49] Hakkim A, Fürnrohr BG, Amann K, Laube B, Abed UA, Brinkmann V, Herrmann M, Voll RE, Zychlinsky A (2010). Impairment of neutrophil extracellular trap degradation is associated with lupus nephritis. Proc Natl Acad Sci USA.

[50] Bratton DL, Henson PM (2011). Neutrophil clearance: when the party is over, clean-up begins. Trends Immunol.

[51] Urban CF, Ermert D, Schmid M, Abu-Abed U, Goosmann C, Nacken W, Brinkmann V, Jungblut PR, Zychlinsky A (2009). Neutrophil extracellular traps contain calprotectin, a cytosolic protein complex involved in host defense against Candida albicans. PLoS Pathog.

[52] Buchanan JT, Simpson AJ, Aziz RK, Liu GY, Kristian SA, Kotb N, Feramisco J, Nizet V (2006). DNase expression allows the pathogen group A Streptococcus to escape killing in neutrophil extracellular traps. Curr Biol.

[53] Berends ET, Horswill AR, Haste NM, Monestier M, Nizet V, von Köckritz-Blickwede M (2010). Nuclease expression by Staphylococcus aureus facilitates escape from neutrophil extracellular traps. J Innate Immun.

[54] Xu J, Zhang X, Pelayo R, Monestier M, Ammollo CT, Semeraro F, Taylor FB, Esmon NL, Lupu F, Esmon CT (2009). Extracellular histones are major mediators of death in sepsis. Nat Med.

[55] Ueki S, Melo RC, Ghiran I, Spencer LA, Dvorak AM, Weller PF (2013). Eosinophil extracellular DNA trap cell death mediates lytic release of free secretion-competent eosinophil granules in humans. Blood.

[56] Bernard GR, Vincent JL, Laterre PF, LaRosa SP, Dhainaut JF, Lopez-Rodriguez A, Steingrub JS, Garber GE, Helterbrand JD, Ely EW, Fisher CJ (2001). Recombinant human protein C Worldwide Evaluation in Severe Sepsis (PROWESS) study group: efficacy and safety of recombinant human activated protein C for severe sepsis. N Engl J Med.

[57] Abraham E, Reinhart K, Opal S, Demeyer I, Doig C, Rodriguez AL, Beale R, Svoboda P, Laterre PF, Simon S, Light B, Spapen H, Stone J, Seibert A, Peckelsen C, De Deyne C, Postier R, Pettilä V, Artigas A, Percell SR, Shu V, Zwingelstein C, Tobias J, Poole L, Stolzenbach JC, Creasey AA, OPTIMIST Trial Study Group (2003). Efficacy and safety of tifacogin (recombinant tissue factor pathway inhibitor) in severe sepsis: a randomized controlled trial. J Am Med Assoc.

[58] Jaimes F, De la Rosa G, Arango C, Fortich F, Morales C, Aguirre D, Patiño P (2009). Unfractioned heparin for treatment of sepsis: a randomized clinical trial (the HETRASE Study). Crit Care Med.

[59] Warren BL, Eid A, Singer P, Pillay SS, Carl P, Novak I, Chalupa P, Atherstone A, Pénzes I, Kübler A, Knaub S, Keinecke HO, Heinrichs H, Schindel F, Juers M, Bone RC, Opal SM (2001). KyberSept Trial Study Group: Caring for the critically ill patient. High-dose antithrombin III in severe sepsis: a randomized controlled trial. J Am Med Assoc.

[60] Esmon CT, Xu J, Lupu F (2011). Innate immunity and coagulation. J Thromb Haemost.

[61] Rapaport SI, Rao LV (1995). The tissue factor pathway: how it has become a “prima ballerina. Thromb Haemost.

[62] Aleman MM, Gardiner C, Harrison P, Wolberg AS (2011). Differential contributions of monocytes- and platelet-derived microparticles towards thrombin generation and fibrin formation and stability. J Thromb Haemost.

[63] Massberg S, Grahl L, von Bruehl ML, Manukyan D, Pfeiler S, Goosmann C, Brinkmann V, Lorenz M, Bidzhekov K, Khandagale AB, Konrad I, Kennerknecht E, Reges K, Holdenrieder S, Braun S, Reinhardt C, Spannagl M, Preissner KT, Engelmann B (2010). Reciprocal coupling of coagulation and innate immunity via neutrophil serine proteases. Nat Med.

[64] Levi M (2008). The coagulant response in sepsis. Clin Chest Med.

[65] Fuchs TA, Brill A, Duerschmied D, Schatzberg D, Monestier M, Myers DD, Wrobleski SK, Wakefield TW, Hartwig JH, Wagner DD (2010). Extracellular DNA traps promote thrombosis. Proc Natl Acad Sci USA.

[66] Brinkmann V, Zychlinsky A (2012). Neutrophil extracellular traps: is immunity the second function of chromatin?. J Cell Biol.

[67] Kambas K, Mitroulis I, Apostolidou E, Girod A, Chrysanthopoulou A, Pneumatikos I, Skendros P, Kourtzelis I, Koffa M, Kotsianidis I, Ritis K (2012). Autophagy mediates the delivery of thrombogenic tissue factor to neutrophil extracellular traps in human sepsis. PLoS One.

[68] Caudrillier A, Kessenbrock K, Gilliss BM, Nguyen JX, Marques MB, Monestier M, Toy P, Werb Z, Looney MR (2012). Platelets induce neutrophil extracellular traps in transfusion-related acute lung injury. J Clin Invest.

[69] von Brühl ML, Stark K, Steinhart A, Chandraratne S, Konrad I, Lorenz M, Khandoga A, Tirniceriu A, Coletti R, Köllnberger M, Byrne RA, Laitinen I, Walch A, Brill A, Pfeiler S, Manukyan D, Braun S, Lange P, Riegger J, Ware J, Eckart A, Haidari S, Rudelius M, Schulz C, Echtler K, Brinkmann V, Schwaiger M, Preissner KT, Wagner DD, Mackman N, Engelmann B, Massberg S (2012). Monocytes, neutrophils, and platelets cooperate to initiate and propagate venous thrombosis in mice in vivo. J Exp Med.

